# Synchronous distance teaching of radiology clerkship promotes medical students’ learning and engagement

**DOI:** 10.1186/s13244-021-00984-w

**Published:** 2021-03-25

**Authors:** Ali Alamer, Fawaz Alharbi

**Affiliations:** Department of Radiology, College of Medicine, Qassim University, Buraidah, 6655-51452 Saudi Arabia

**Keywords:** Radiology, Undergraduate, Medical education, E-learning, Distance learning

## Abstract

**Background:**

The COVID-19 pandemic has impacted education in myriad ways, primarily leading to an abrupt paradigm shift in teaching and learning practices towards distance learning. The study aims to assess the effectiveness of teaching radiology to undergraduate medical students using synchronous distance learning compared to traditional on-campus learning through exploring students’ perceived satisfaction and concerns. Students’ perceptions were correlated with their attendance, grades, and frequency of technical difficulties.

**Methods:**

The study was designed as an observational study involving fourth-year medical students (2019/2020) from two institutions. The cohort students were exposed to traditional learning, distance learning, or both. Students completed an online self-administered questionnaire concerning their perceptions of distance learning. Students’ attendance, engagement, technical difficulties, and post-clerkship knowledge assessments were analyzed using descriptive and inferential statistics.

**Results:**

A total of 145 participants completed the clerkship using the following strategies: traditional learning (*n* = 66), both traditional and distance learning (*n* = 67), and distance learning alone (*n* = 12). The most important result indicates that the abrupt transition to distance learning was well perceived. Most students preferred distance learning over traditional learning in the radiology clerkship (*p* = .05). During the synchronous sessions, student attendance was high, reaching to 100%. Technical difficulties were limited (1.9%), and they did not affect learning.

**Conclusion:**

Synchronous distance teaching promotes learning, interaction, and enjoyment in undergraduate radiology education, and it can be as effective as traditional on-campus learning. The technical difficulties encountered, although they were limited, can be overcome by recording the synchronous sessions.

## Key points

- The abrupt transition from traditional to completely distance learning was well perceived.
- Distance learning in undergraduate radiology education, if augmented with synchronous interaction, can be as effective as traditional on-campus learning.
- Synchronous distance teaching was the preferred mode of learning as it promotes knowledge gain, interaction, and enjoyment.
- The use of recorded sessions proved to be a source for knowledge gain and a solution for technical difficulties.

## Background

In December 2019, cases of serious illness causing primarily pneumonia and subsequent mortality of unknown etiology appeared in Wuhan; the capital city of Hubei province in central China [1]. The causative coronavirus was then identified by the Chinese Center for Disease Control and Prevention (CCDC), and the associated disease was then called COVID-19 by the World Health Organization (WHO) in February 2020 [2]. In March 2020, the WHO declared COVID-19 outbreak a pandemic following its global spread [3]. Up to date, more than 1.5 million people have died from COVID-19 worldwide [4].

As of March 2020, several countries decided to temporarily suspend attendance in schools and universities until further notice. The decision was based on the preventive and precautionary measures advised by health authorities worldwide as part of their efforts to control COVID-19 spread. However, to support learning during the period of suspension, governments and educational authorities resorted to deploying electronic (e)-learning solutions. E-learning as a generic term can be defined as the use of information technologies to develop and enhance skills and knowledge gain [5, 6]. Generally, there are two common modes of e-learning: on-campus computer-assisted learning and online distance learning [7]. Distance learning can be synchronous or asynchronous [5]. In synchronous distance learning, students participate in virtual classes or live webinars at the same time with their teachers and peers. Class interactions happen in real-time unlike the case in asynchronous distance learning which does not need live classroom participation. In asynchronous learning, students and teachers work through course material at different times.

Driven by technological advancement, e-learning has become a fundamental part of medical education, particularly in radiology as a digital specialty which presents opportunities for educational innovation in the form of e-learning resources [6, 8]. Zafar et al. [6] review on using e-learning in the field of radiology education at undergraduate level reported an increase in implementing blended learning environments. However, during the current COVID-19 pandemic, there has been an emergency paradigm shift in teaching and learning globally which caused a complete transformation into distance learning. Due to the abrupt change to distance learning, there has been an inconsistency in the way distance learning has been implemented in different medical schools worldwide. Within this context, the Department of Radiology at our institution has completely transformed its two-credit-hour radiology clerkship conducted for the fourth-year medical students from traditional on-campus learning to entirely distance learning. The usual traditional on-campus radiology sessions were replaced by online synchronous sessions using a pre-existing learning management system (LMS).

The application of e-learning has been previously evaluated in teaching radiology [6, 9]. However, only two articles discussed the use of synchronous online learning to teach radiology to undergraduate medical students prior to COVID-19 pandemic [10, 11]. A recent study has researched the effectiveness of synchronized online learning from the perspectives of undergraduate medical students, however, our study differs in its aims, methods, and particular focus on radiology clerkship experiences [12]. To the best of our knowledge, we are not aware of any published study which specifically examines students’ perceptions of the effectiveness of using synchronized distance learning in radiology at the undergraduate level in a standalone clerkship programme compared to using traditional on-campus learning. Furthermore, there is no sufficient data on experiencing technical issues during teaching radiology through attending synchronous sessions and their impact on the intended learning outcomes. Therefore, our study aims to assess the effectiveness of teaching radiology to undergraduate medical students using online synchronous sessions. The effectiveness of distance learning was assessed using Kirkpatrick’s model on two levels: reaction and learning [13]. The study explores students’ perceptions of their experiences, particularly, their perceived satisfaction and concerns regarding the abrupt transition to distance learning which might be used to improve the effectiveness of teaching radiology using distance learning, reveal some obstacles, and formulate recommendations or solutions. Achieving learning outcomes, experiencing technical difficulties, and the use of recorded sessions which is, presumably, a solution for the anticipated and unavoidable technical difficulties are also discussed.

## Methods

### Design of teaching methods before COVID-19 pandemic

Radiology is a compulsory clerkship for fourth-year students of our five-year Bachelor of Medicine and Surgery programme. By the end of the clerkship, students should be competent to: (1) request the radiological investigation of choice to solves the patient’s problem; (2) diagnose common medical conditions by interpreting different imaging modalities with specific emphasis on emergency cases; and (3) be aware of safety and hazards in radiology.

Prior to COVID-19 pandemic, the radiology clerkship activities included attending on-campus lectures which were taught over the semester, normally two hours per week, giving a total of 24 contact hours. Topics were based on different radiological subspecialties; and they were presented by subspecialized radiologists. A few days prior to each lecture, the students receive several cases with clinical scenarios and radiological images. The cases were designed to challenge the students to choose the appropriate radiological investigation and/or to localize the abnormality on radiological images. *Imaging for Students* (David A Lisle, 2012) and Radiopedia (www.radiopedia.org) were suggested to students as learning resources. During the lecture, mixed didactic and case-based approach was utilized to address the knowledge gap and areas of weaknesses in ordering radiological studies and interpretation skills. Utilization of e-learning was limited to simply reviewing the schedule, course announcements, and downloading lecture notes or scripts.

The clerkship programme evaluation included testing the students twice in the mid and end of the clerkship. The testing protocol consisted of both multiple-choice questions (MCQ) and objective structured practical examination (OSPE). The OSPE is a practical exam to assess students’ radiological image interpretation skills of common and emergency cases. The tutors construct the OSPE items based on a blueprint in accordance with the clerkship’s objectives. During the exam, students go through a series of cases, presented in an automated manner (70 s for each item) using overhead projector. Each case has a radiological image with a relevant clinical scenario followed by a specific question.

### Design of teaching methods during the COVID-19 pandemic

As a result of COVID-19 pandemic and its precautionary measures, our department has completely transformed the way radiology clerkships were taught from traditional on-campus learning to entirely distance learning. The transformation has been applied after the 8th week of the 2nd semester during 2019/2020 academic year. The topics and instructional approach were generally unchanged since the 1st semester. A total of 14 contact hours had been conducted using on-campus lectures. As a result of time constraints, the remaining scheduled ten contact hours were reduced to eight hours; and were delivered using online synchronous sessions via Blackboard (Blackboard Inc., Washington, D.C., United States). Blackboard is one of the largest educational platforms which are accessible for all registered students via internet access using their individual usernames and passwords. It offers an online course platform with several features such as announcements, virtual classrooms, uploading learning materials, assignments, and assessments.

As soon as a distance learning-based curriculum was adopted, students at our department received multiple announcements describing the new changes, in particular how to access the virtual sessions to ensure a smooth transition. The in-charge tutor is responsible for creating a virtual session via Blackboard Collaborate. Blackboard Collaborate is a synchronous online tool that allows tutors and their students to communicate in real-time via a virtual classroom. Tutors access the session as moderators who can share files for their presentations. Students are given a participant role which allows them to interact with the tutor, as for example, asking or answering questions verbally or via chat (as per rules set by the tutor). Blackboard Collaborate provides a report by the end of each session which gives an overview of when students joined and left the session, and how many times they re-joined the session. It also shows how long students were present in the session. Tutors are instructed to turn recording on in sessions to allow students to view the recordings at any time. Although the course forum is not activated, all tutors at our institution have been instructed to maintain frequent and effective communications with their students, ideally through the institutional email address published in the Blackboard address book.

### Faculty development

As the successful implementation of distance learning requires rapid engagement in faculty development, particularly with such an abrupt transition we had, we conducted two-day faculty development virtual sessions on Blackboard Collaborate. The sessions provided a detailed explanation of the design of the Blackboard and its features, in particular how to create a virtual classroom. The sessions were presented by an e-learning experienced instructor, who is also a member of the digitalization committee at our institution. A total of eight participants (80% of the faculty tutors) attended these sessions. Training on Zoom (Zoom Video Communications Inc., San Jose, California, United States) was also performed as a backup for Blackboard in case of technical difficulties during the scheduled live streaming with students. Furthermore, the deanship of e-learning and distance education at our institution has arranged additional training programmes for the faculty members on using and managing virtual classrooms through Blackboard. Tutors were also instructed to submit a report to the course organizer at the end of their educational activities including date, time, and duration of the session as well as their overall satisfaction on its implementation. Furthermore, the tutors received post-clerkship survey to capture their perceptions of radiology distance learning. The tutors specify their level of agreement to various statements using a five-point Likert scale.

### Participants

All fourth-year medical students (*n* = 133) from 2019/2020 academic year who underwent a compulsory radiology clerkship were included in the study. Selection bias could thus be excluded. Students were randomly allocated by the office of deanship for educational affairs into two groups. The first one, Group (A) completed the radiology clerkship during the 1st semester via traditional on-campus face-to-face sessions (*n* = 66). The second one, Group (B) completed the radiology clerkship during the 2nd semester using both traditional on-campus face-to-face sessions prior to COVID-19 pandemic and online synchronous sessions during the COVID-19 pandemic (*n* = 67). Attending online synchronous sessions was voluntary unlike attending on-campus face-to-face sessions and the clerkship assessment tools which were compulsory.

### Assessment of learning outcomes

Online assignments and exams were used as tools to assess students’ achievement of clerkship learning outcomes during the COVID-19 pandemic (Group B). In relation to assignments (40% of the total marks), students had to do four homework assignments. Three assignments focused on topics which were previously presented in the virtual sessions. The fourth assignment focused on topics related to the current COVID-19 pandemic with specific emphasis on precautions, role of imaging, and chest imaging findings, which although they were beyond the scope of this clerkship, they were considered important during this time. The assignments were submitted online via Blackboard.

As for exams (50% of the total marks), after taking trial quizzes using Blackboard and Microsoft Forms (Microsoft Corporation Inc., Redmond, Washington, United States), students took four online MCQ-type exams using Microsoft Forms, which was students’ preferred choice of an assessment tool. Furthermore, one virtual oral exam (10% of the total marks) was also performed, with specific emphasis on assessing the interpretation skills of students in emergency cases. The questions were reviewed by all board-certified radiologists at the faculty to confirm validity, reliability and fairness. To prevent bias, randomly selected questions were also reviewed by non-radiologists in the assessment unit at our institution. The overall assessment protocol was approved by the electronic assessment committee which was established at our institution during the COVID-19 pandemic.

### Evaluation of students’ perception

Student feedback surveys are part of the curricular assessment at our institution. Surveys were routinely available on Blackboard for students to fill out online at the end of each clerkship. Feedback from students in Group A (1st semester) and Group B (2nd semester) was reviewed for quality assurance purposes. Students in Group B received an additional modified survey which specifically addressed distance learning compared to traditional on-campus learning in radiology clerkship. The survey’s items were developed based on a thorough literature review which provides an overview of the constructs of interest that has been defined in prior studies [6, 9]. The draft survey items have undergone a pre-test and further refined by interviewing students who represent the target participants [14].

The final survey was then designed as web-based using SurveyMonkey (SVMK Inc., San Mateo, CA, United States). It was composed of 18 questions categorized into four subsections, each addressing different aspects. The first section consisted of demographic-related questions. The second section was aimed at evaluating whether the students had a prior experience with distance learning. The third section addressed technical accessibility, including the preferred device for distance learning and the reasons for technical difficulties during synchronous sessions. In the last section, the students were presented with various statements regarding their perceptions of distance learning in radiology clerkship compared to traditional on-campus learning which include: the effect of abrupt transition from traditional to distance learning, causes of absenteeism in online synchronous sessions, and using recorded sessions. A mixture of questions’ types was used in the survey to capture students’ perceptions including: a five-point Likert scale, ‘Yes’ or ‘No’, MCQ, and open-ended questions. Students were also given the opportunity to enter free-text comments. The survey was sent out to students via email at the end of the clerkship and before announcing the final clerkship results to avoid bias. Students were informed that participation was voluntary and anonymous without incentives or rewards. The main aims of the study were also explained to students.

### Modification of distance learning at another institution

There is an academic collaboration between our institution and another regional medical college, in which the compulsory fourth year radiology clerkship is conducted by our faculty. A total of 24 contact hours were presented to 12 medical students in a one-week duration via distance learning (Group C). The topics and contents are basically unchanged. However, the instructional approach was modified to include separate didactic sessions (18 h) and case-based sessions (6 h). During the case-based sessions, students discussed radiology cases and practically applied knowledge learned from prior didactic sessions. Contrary to Group B, the cases were not sent to the students prior to the sessions and were utilized for assessment purposes. The testing protocol consisted of continuous assessment during the lectures and live discussion sessions (48%), three homework assignments (22%), one online virtual oral exam (15%), and one MCQ-type online exam (15%). Students were also invited to participate in the aforementioned survey distributed to Group B to understand their perceptions of distance learning.

### Data analysis

The collected data from the sessions’ reports, students’ grades and surveys were registered as a Microsoft Excel spreadsheet (Microsoft Corporation, Redmond, Washington). The descriptive statistics were calculated using Excel data analysis tool. Students’ perceptions were correlated with their grades and attendance for reliability purposes. Chi-square test of independence was used to determine if a statistically significant relationship exists between two categorical variables. A two-sample t-test was also conducted for comparison between different means. A *p* value of ≤ 0.05 was considered to be statistically significant.

## Results

### Participants

A total of 145 medical students were enrolled in the radiology clerkships during 2019/2020 academic year. Group A students (*n* = 66) attended the traditional on-campus clerkship prior to COVID-19 pandemic, including 45 males (68.2%) and 21 females (31.8%). Group B students (*n* = 67) was exposed to both learning methods; traditional learning prior to COVID-19 pandemic and distance learning during the COVID-19 pandemic. Group B included 47 males (70.1%) and 20 females (29.9%). Group C included 12 male students who were fully engaged in distance learning.

### Sessions’ attendance

In Group A, the attendance rate of the enrolled students was 89.5%. In Group B, the attendance rate for on-campus sessions was 93.5%. However, the attendance rate has dropped to 60.1% for online synchronous sessions. Finally, the attendance rate for Group C students was 100% (Table 1).

**Table 1:** Attendance rate of traditional (on-campus sessions) and distance learning (online synchronous sessions)

Group	Scheduled contact hours (%)		Attendance of traditional learning (%)	Attendance of distance learning (%)
	Traditional learning	Distance learning		
A	100	0	89.5	0
B	63.6	36.4	93.5	60.1
C	0	100	0	100

Regarding the online synchronous sessions, 63.2% of Groups B and C students attended on time or even earlier; with an average of 4.7 min before the scheduled time. The majority of students (86.3%) were present online for more than 90% of the duration of sessions (the average session duration was 1:07:15). Less than a quarter (22.8%) of students experienced technical difficulties during their sessions. However, technical difficulties affected only 1.9% of the total sessions’ time (Table 2).

**Table 2:** Attendance of online synchronous sessions and technical difficulties

Group	Average time per session	Students’ attendance		Present online for more than 90% of sessions time (%)	Technical difficulties (%)
		On time (%)	Delayed (%)		
B	1:06:04	57.8	42.2	77.0	1.5
C	1:16:06	68.5	31.5	95.2	0.7

### Assessment of learning outcomes

**Fig. 1:**
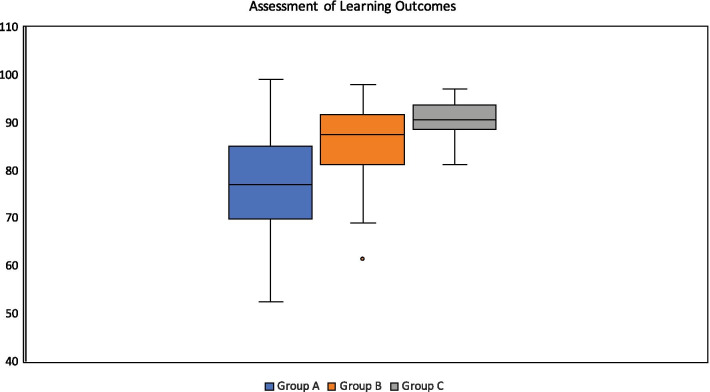
Comparison of boxplots data sets (post-test scores) in between Groups A, B, and C

In Group A, 64/66 students (97.0%) completed the on-campus clerkship exams. While in Group B and C, 79/79 students (100%) completed the online clerkship exams. The post-tests’ mean percentage scores (ratio of correctly answered questions to total number of test questions) were calculated for all groups (Table 3). The overall results of assessments were also displayed in boxplots data sets for all groups (Fig. 1). As the synchronous online sessions’ attendance was optional for students in Group B, there is a significant positive relationship between the number of the attended sessions and the scores of correct answers, *r*(65) = 0.39, *p* < 0.05.

**Table 3:** Mean percentage scores on post-test ± standard deviation (SD) and 95% confidence interval

Group	Modes of learning	Post-test mean % ± SD	95% CI
A	Traditional	77.46 ± 10.21	2.50
B	Traditional and distance	86.48 ± 7.45	1.78
C	Distance	90.23 ± 5.03	2.85

### Students’ perceptions of distance learning

#### Response rate, personal characteristics, and prior experience with distance learning

In total, 81.0% of students in Groups B and C (*n* = 64/79) participated in the survey; 73.4% (*n* = 47) were males, and 26.6% (*n* = 17) were females. Their mean age was 23.38 (± 1.43). The majority of participants (*n* = 50, 78.1%) did not have any prior experience with online synchronous sessions. However, out of 14 students who indicated that they already had prior experience, courses run by the Virtual Medical Academy (www.medicalacademy.org) were specified in 14.3% of responses. None of the students reported using Blackboard for synchronous distance teaching prior to this clerkship.

#### Transition and accessibility

The new and abrupt transition from traditional to distance learning during the COVID-19 pandemic was found to be smooth by 70.3% (*n* = 45) and it did not negatively affect their learning. All participants (100%) agreed that the department’s instructions on how to access the online synchronous sessions via LMS were clear and useful. The majority of students (*n* = 52, 81.3%) accessed the online synchronous sessions using computers, either desktops or laptops. Smartphones and other smart devices such as iPads were used by the students to access the sessions in only 6.2% (*n* = 4) and 12.5% (*n* = 8), respectively.

#### Preference of online synchronous sessions over on-campus face-to-face sessions

As an overall learning experience, the majority of students (*n* = 40, 62.5%) preferred online synchronous sessions, as a mode of learning, over on-campus face-to-face sessions. Based on the analysis of free-text comments, online synchronous sessions were rated to be cost effective, saving time, and efforts (*n* = 15, 23.4%). The high resolution of the radiological images (*n* = 12, 18.8%) and the ability to record sessions (*n* = 11, 17.2%) were also considered as other reasons for preference of online synchronous sessions. On the other hand, a minority of students found on-campus face-to-face sessions more interactive (*n* = 9, 14.1%) due to the positive effects of the teacher's body language and giving students more opportunities to ask questions. Some students (*n* = 9, 14.1%) indicated that they had higher levels of concentration and were less distracted during on-campus face-to-face sessions.

Fewer than half of students (*n* = 29, 45.4%) agreed that they had better knowledge gain when online synchronous sessions were used compared to on-campus face-to-face sessions, while 25.0% were neutral regarding having better knowledge gain and 29.6% disagreed with the statement (Table 4). Students who indicated that they had better knowledge gain when online synchronous sessions were used attributed their belief to two main reasons: attending virtual sessions in the comfort of their home (30.5%) and the advantage of using recorded sessions (30.5%). More than half of students (*n* = 33, 51.6%) agreed that using online synchronous sessions has facilitated more interactions between students and tutors. The reasons were attributed to the stress-free environment (83.8%), improved concentration (59.5%), encouragement by tutors (48.7%), and encouragement by the interactions of other students (46.0%). The majority of students (*n* = 40, 62.5%) found online synchronous sessions more enjoyable than on-campus face-to-face sessions.

**Table 4:** Students’ preference of mode of learning in radiology clerkship

	Online synchronous session (%)	On-campus face-to-face sessions (%)	*p* value
Overall students’ preference	62.5	37.5	.05
Knowledge gain	60.4	39.6	.15
Student-tutor interaction	61.1	38.9	.10
Enjoyment	74.1	25.9	< .001
Less stressful	73.8	26.2	.002

Students who chose “I do not know” were excluded from the calculations in this table

#### Technical difficulties, attendance and use of recorded sessions

The results indicate that more than half of students (*n* = 33, 51.6%) reported no technical difficulties during online synchronous sessions that might have negatively interfered with their understanding of the given topics. Other students rated their experience with technical difficulties as rarely (37.5%), sometimes (7.8%) and often/always (3.1%). All students who experienced technical difficulties during virtual sessions attributed the reason to network-related issues.

Regarding attendance, based on students’ perspectives, the three leading causes of absenteeism from the online synchronous sessions were time-related (54.3%), internet-related (45.7%), or due to the availability of recorded sessions (31.4%). Based on the survey results, 47.6% of students always reviewed the recorded sessions while 22.2% often, 15.9% sometimes, 4.8% rarely, and 9.5% never reviewed the recorded sessions. Of those students who took advantage of recorded sessions, 89.3% used them to prepare for exams, 53.6% reviewed the sessions at a later time as they lost concentration during the synchronous sessions, 23.2% used them because they were unable to attend the synchronous sessions, and 17.9% used them because they had technical difficulties during the synchronous sessions.

### Faculty’s perceptions of distance learning

**Fig. 2:**
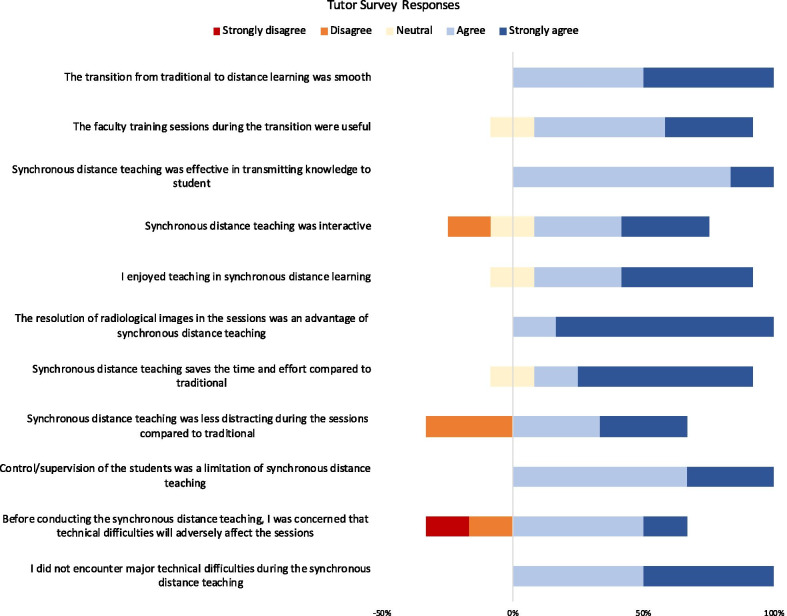
Tutor survey responses

The tutors (*n* = 6) have two to nine years of experience in radiology teaching to undergraduate medical students. None of the tutors reported using Blackboard for synchronous distance teaching prior to this clerkship. Tutor survey responses are listed in Fig. 2.

## Discussion

In 2000, Davison et al. [15] published the first attempt to digitalize the radiology clerkship for undergraduate medical students. Since then, e-learning in radiology has been rapidly evolving [6, 9]. Radiology, as a digital specialty, has also experienced a tremendous technological advancement over the past decades such as applying Picture Archiving and Communication System (PACS), making radiology a rich environment for innovative e-learning [16, 17]. As the current COVID-19 pandemic has speeded-up the changes in teaching strategies from traditional to distance learning [18], our department has quickly adapted with such changes and transformed its traditional radiology teaching into virtual synchronous sessions via a pre-existing LMS. Although the majority of our students and tutors did not have prior experience with synchronous distance teaching, the new and abrupt transition from traditional to distance learning was well perceived. Similar to the results of previous studies [11, 12], students in our study enjoyed attending online synchronous sessions and rated their experience highly. Moreover, the transition to distance learning did not negatively affect achieving the learning outcomes of the radiology clerkship. We found that clear and timely instructions and communications, in addition to, faculty training were the major determinants of smooth transition of learning strategies as all students and faculty found it clear and useful.

Upon evaluating nuclear medicine e-learning module, Dissel et al. [19] reported that the majority of students had prior experience with e-learning in general, reaching up to 88%. However, a minority of our students had prior experience with virtual synchronous sessions in radiology as a subtype of e-learning, which is in line with the results of a prior study [11]. To the best of our knowledge, only two articles discussed the use of online synchronous sessions to teach radiology to undergraduate medical students prior to COVID-19 pandemic [10, 11]. For example, Tachakra et al. [10] documented improved students’ comprehension with videoconferences in teaching radiological series of skeletal injuries. Lorenzo-Alvarez et al. [11] explored the potential use of virtual reality for undergraduate radiology education, and they found it feasible and well-received by students. The attendance to their radiology sessions in the virtual world was mandatory, which reached up to 88.6% [11]. In our study, the attendance rate for online synchronous sessions was significantly higher (*p* < 0.001) in Group C (100%) as compared to Group B (60.1%), which is clearly explained by the optional attendance in the latter. Although attendance was compulsory for students in Groups A and C, the attendance rate was higher in Group C (100% compared to 89.5%). The high attendance rate in Group C may have been motivated by allocating two marks for active class participation in each synchronous session that students attend.

Despite advancement in smartphones technology, the majority of our students accessed the online synchronous sessions using their computers. This result can be explained by better accessibility of Blackboard Collaborate features, and better viewing of radiological images. Dissel et al. [19] found that almost all medical students in their cohort sample have their own computers with internet access, and the majority of the students felt they have good or very good computer skills when accessing the radiology e-learning module.

The advantages of using distance learning over traditional on-campus learning have been thoroughly discussed in the literature, including for example, its cost effectiveness and the opportunity for learning at any time and place at students’ convenience [9, 17]. However, there are possible limitations of e-learning such as experiencing technical difficulties, in particular during online synchronous sessions, which might affect learning outcomes [20]. Less than a quarter (22.8%) of the students in this study experienced minor technical difficulties during online synchronous sessions. However, these difficulties only affected 1.9% of the total sessions’ duration, which is negligible. Not surprisingly, about half (45.6%) of these technical difficulties were on the first day of transition from traditional to distance learning, which can be explained by the lack of prior experience and proper setups. Having technical difficulties in online synchronous undergraduate radiology education were similarly reported in only one study [11]. Based on students’ perception, 9% of participants in the study of Lorenzo-Alvarez et al. [11] reported technical difficulties such as network-related issues. The study concluded that although such technical difficulties can limit proper interaction, they will progressively decline due to technological advancement.

Reviewing the literature indicates that there is no sufficient published data on the evaluation of distance learning in undergraduate radiology education, in particular, whether distance learning could completely replace traditional learning based on students’ perceptions. Most of the studies focus on using asynchronous distance learning to complement and enhance traditional learning rather than to investigate whether distance learning can replace traditional learning. For these reasons, the majority of students preferred a blended learning approach for radiology teaching over e-learning alone [10, 21–23]. However, to compare the effectiveness of traditional and distance learning, using synchronous interaction in online learning environments should be considered, which this study intended to assess. The results of our study indicate that the overall distance learning experience in radiology was well-perceived by our students, which is in line with most recent studies [9, 12, 17]. Furthermore, the e-learning tools we used in our project were favored by the students over traditional learning (*p* = 0.05) due to saving cost, time, and effort. Improved radiological image resolution in the virtual sessions compared to classroom projectors was another reason for favoring distance learning by both students and tutors, which is of particular importance for radiology teaching.

Regarding knowledge gain, the results indicate that distance learning was rated higher than traditional learning as students attended the sessions in their comfort zone, in addition to the perceived benefits of recorded sessions. However, such preference was not statistically significant, probably because some students believe traditional learning is associated with better concentration and is augmented by teacher's body language. The knowledge gain can be also partially validated by post-test with higher mean scores in Group C, followed by Groups B and A. The differences between these means achieved statistical significance (*p* < 0.001). To eliminate confounding factors and biases, the results show that there is a significant positive relationship between the optional attendance for students in Group B and the results of assessments of the attended sessions (*p* < 0.05), which further validate improved knowledge gain. The knowledge gain in distance learning for radiology contents was also previously proved in prior studies using pre and post-tests [24–27].

Using the interactive learning approach in radiology, ranging from simple annotation of radiological images to face-to-face interaction, has been already evaluated in prior studies [8, 28]. In our study, most of the students rated distance learning higher than traditional learning with regard to student-tutor interaction. The students attributed this to improved concentration in distance learning, being encouraged by others to ask questions, and having a stress-free environment. However, such preference was not statistically significant possibly because some students did not have the proper setups for interaction, the students did not know how to use the interaction tools in Blackboard, or because they felt the sessions’ time was short.

The engagement of medical students and its effect on learning have been well documented in the literature, which can be promoted by accessing enjoyable contents and presentations [29]. In our study, students’ feedback was positive in relation to enjoyment in distance learning, which is comparable to prior studies [19, 30]. Davison et al. [15] reported 95% of students in radiology clerkship enjoyed the online approach more than traditional one, which is in parallel with our results. In our study, the students enjoyed distance learning more than traditional learning, which achieved statistical significance (*p* < 0.001). This positive feedback was validated by 63.2% of students who attended online sessions on time and 86.3% who were present for more than 90% of the duration of sessions. The engagement of Group C students (95.2%) was higher than Group B (77.0%). However, this result may have been influenced by the aforementioned two marks allocated for active class participation in each online session. Moreover, students in our study perceived distance learning to be less stressful than traditional learning, which achieved statistical significance (*p* = 0.002). Such perception is another contributory factor for enjoying and engaging with distance learning.

Using pre-recorded video lectures in e-learning has been previously evaluated for teaching radiological contents to medical students [8, 21, 23, 27, 31]. However, to the best of our knowledge, there are no sufficient published data on the use of recorded live virtual sessions. We suggest that recorded sessions might overcome technical difficulties where students can review the sessions at their convenience. In our study, having recorded sessions was well perceived by students. Students indicated that the recorded sessions were very useful to prepare for exams. Some students used the recorded sessions when they were absent, or when they had technical difficulties during synchronous sessions. On the other hand, some students (31.4%) found having recorded sessions a good reason for absenteeism from synchronous sessions.

## Limitations

We acknowledge some limitations of our study. The direct comparison between distance and traditional learning based on outcomes was presumably biased by time frame as we tested the traditional group in the 1st semester and the online group in the 2nd semester. Such bias can be solved by adopting a crossover technique, where two different groups are assessed simultaneously, and are exposed to the same topics and same tests. Furthermore, the assessment of outcomes and knowledge gain was limited by a lack of pre-test in our study design. Such limitation was due to the abrupt transition from traditional to distance learning during the COVID-19 pandemic. Some students missed the advantage of teacher’s body language in the virtual sessions, which can be solved by using webcam video in virtual sessions. However, in this study, webcams were not used due to the assumption that they could be a source of distraction for teachers during live streaming, and that using them may result in network-related problems. These assumptions require further validation. Finally, based on the inconsistency in distance learning during the COVID-19 pandemic, recruiting more centers is recommended, firstly to increase the number of participants, and secondly to evaluate their newly adopted techniques in distance teaching of radiology clerkship.

## Conclusions

This study is in line with prior published studies which explored the feasibility and effectiveness of distance learning in radiology for undergraduate medical students. The results indicate that distance learning, if augmented with online synchronous interaction, can be as effective as traditional on-campus learning, and it can even be used as an alternative to traditional learning. However, distance learning like other learning strategies is not immune to obstacles and limitations such as primarily experiencing technical difficulties. Luckily, such technical difficulties will progressively decline in the near future with advancement in technologies.

Successful implementation of distance learning in radiology requires a preexisting LMS with a reliable and user-friendly virtual classroom solution. Training of tutors and participants on LMS features and accessibility is crucial for clerkship success. Furthermore, the LMS’s announcements tools are essential and should be optimized to help abrupt transition and achieve learning strategies. The use of recorded sessions proved to be a source for knowledge gain and a solution for technical difficulties that students may encounter.

## Data Availability

The datasets used and analyzed during the current study are available from the corresponding author on reasonable request.

## Abbreviations

CCDC: Chinese Center for Disease Control and Prevention
COVID-19: Coronavirus disease 2019
E-learning: electronic learning
LMS: learning management system
MCQ: multiple-choice question
OSPE: objective structured practical examination
PACS: picture archiving and communication system
WHO: World Health Organization
